# Use of Lot Quality Assurance Sampling to Ascertain Levels of Drug Resistant Tuberculosis in Western Kenya

**DOI:** 10.1371/journal.pone.0154142

**Published:** 2016-05-11

**Authors:** Julia Jezmir, Ted Cohen, Matteo Zignol, Edwin Nyakan, Bethany L. Hedt-Gauthier, Adrian Gardner, Lydia Kamle, Wilfred Injera, E. Jane Carter

**Affiliations:** 1 Stanford Medical School, Stanford, California, United States of Amercia; 2 Academic Model Providing Access to Healthcare (AMPATH), Eldoret, Kenya; 3 Department of Epidemiology of Microbial Disease, Yale School of Public Health, New Haven, Connecticut, United States of America; 4 Global TB Programme, TB Monitoring and Evaluation, World Health Organization, Geneva, Switzerland; 5 Department of Global Health and Social Medicine, Harvard Medical School, Boston, Massachusetts, United States of America; 6 Indiana University School of Medicine, Indianapolis, Indiana, United States of America; 7 Alpert School of Medicine at Brown University, Providence, Rhode Island, United States of America; Indian Institute of Science, INDIA

## Abstract

**Objective:**

To classify the prevalence of multi-drug resistant tuberculosis (MDR-TB) in two different geographic settings in western Kenya using the Lot Quality Assurance Sampling (LQAS) methodology.

**Design:**

The prevalence of drug resistance was classified among treatment-naïve smear positive TB patients in two settings, one rural and one urban. These regions were classified as having high or low prevalence of MDR-TB according to a static, two-way LQAS sampling plan selected to classify high resistance regions at greater than 5% resistance and low resistance regions at less than 1% resistance.

**Results:**

This study classified both the urban and rural settings as having low levels of TB drug resistance. Out of the 105 patients screened in each setting, two patients were diagnosed with MDR-TB in the urban setting and one patient was diagnosed with MDR-TB in the rural setting. An additional 27 patients were diagnosed with a variety of mono- and poly- resistant strains.

**Conclusion:**

Further drug resistance surveillance using LQAS may help identify the levels and geographical distribution of drug resistance in Kenya and may have applications in other countries in the African Region facing similar resource constraints.

## Introduction

Africa is one of the two regions worldwide most severely affected by tuberculosis (TB). According to the World Health Organization’s (WHO) most recent estimates, in 2013, countries in the African Region had the highest TB incidence rates with a regional average of 280 cases/100,000 people/year but as high as 860 cases/100,000 people/year in some countries. An estimated 29% of the world’s TB cases occur in the African Region, where high HIV prevalence helps to drive the TB epidemic [1]. In Kenya, HIV prevalence is estimated at 5.6–6.3% nationally, with significant regional variation [2,3]. In 2013, 38% of TB patients were co-infected with HIV [1].

Despite efforts at local and national levels throughout the region to strengthen TB control, there is ongoing concern about the emergence of multidrug resistant TB (MDR-TB) and extensively drug resistant TB (XDR-TB). In 2013, the WHO estimated that in the African Region among notified pulmonary TB cases, there were 25,000 (2,100–52,000) cases of MDR-TB among treatment-naive individuals, defined as patients not previously treated for TB, and 18,000 (28–37,000) cases among retreatment patients [1]. With high HIV prevalence, drug resistant TB is particularly concerning as HIV positive patients are more likely to present with sputum smear negative TB, which is more challenging to diagnose than sputum smear positive TB, and may thus be more likely to face higher mortality.

Direct efforts to measure the burden of MDR-TB throughout the region have been limited. Current WHO guidelines recommend continuous surveillance through drug sensitivity testing (DST) of all previously treated and treatment-naïve TB patients. Where such capacity is not available, the WHO advises conducting special national surveys to determine the drug resistance burden. On average, these surveys have sample sizes of 1000–1500 patients and cost around $250,000-$300,000 [4]. Due to resource constraints, there is great variability in the type and frequency of surveillance in each country. Across the region, less than 1% of treatment naïve patients and less than 8% of retreatment patients were screened for drug resistance. While 75% of the estimated 43,000 patients with MDR-TB were notified in the region, 80% of those notified were from South Africa, indicating that there remains a substantial pool of prevalent MDR-TB but limited capacity for diagnosis and treatment throughout the rest of the region [1].

Kenya is one of several countries in the region whose drug resistance estimates are outdated. The most recent national, population-representative survey of drug resistant TB was published in 1995, at which time no cases of MDR-TB were identified [1]. Kenya received Global Fund Round 9 support for another national drug resistance survey, which, at the time of writing in 2016, is still in progress. The complexity of conducting drug resistance surveys in countries with limitations in resources and laboratory capacity points to a need to simply the operations of conducting surveys to yield more locally relevant and frequent drug-resistance information [5–7].

While limited information exists about the burden of drug resistant TB in Kenya, even less is known about its geographic distribution within the country. Currently, most MDR-TB patients have been identified in Nairobi likely due to their proximity to Kenya’s Central Reference Laboratory (CRL). However, Kenya’s Division of Leprosy, TB, and Lung Disease (DLTLD) suspects an elevated incidence of drug resistant TB may exist in western Kenya. The region reported two confirmed patients with XDR-TB in 2007, and one of whom was likely the result of primary XDR-TB transmission based on contact investigation [8].

In this study, we employed Lot Quality Assurance Sampling (LQAS) as a method for conducting a targeted drug-resistance survey to determine whether the risk of MDR-TB among treatment-naïve individuals was elevated above a pre-specified threshold [9]. The study was conducted in two different settings in western Kenya: one rural area suspected of a higher risk of MDR-TB and one urban area.

## Methods

### Study sites and sampling

Study sites were selected in collaboration with the DLTLD and the Academic Model Providing Access to Healthcare (AMPATH), a partner of the Ministry of Health supporting comprehensive HIV and chronic disease care in western Kenya. AMPATH is a collaboration between Moi University School of Medicine, the Moi Teaching and Referral Hospital (MTRH), and a group of North American academic medical centers. The two study settings were selected based on predefined geographic areas, the catchment areas of the clinics within the setting, and the population densities of each region.

For the urban setting, we worked with MTRH, located in Eldoret, Kenya, a geographic region with high population density. MTRH is a tertiary referral center, serves as the largest HIV treatment outpatient center in the region, and provides primary care for 300,000 urban patients in Eldoret.

**Fig 1:**
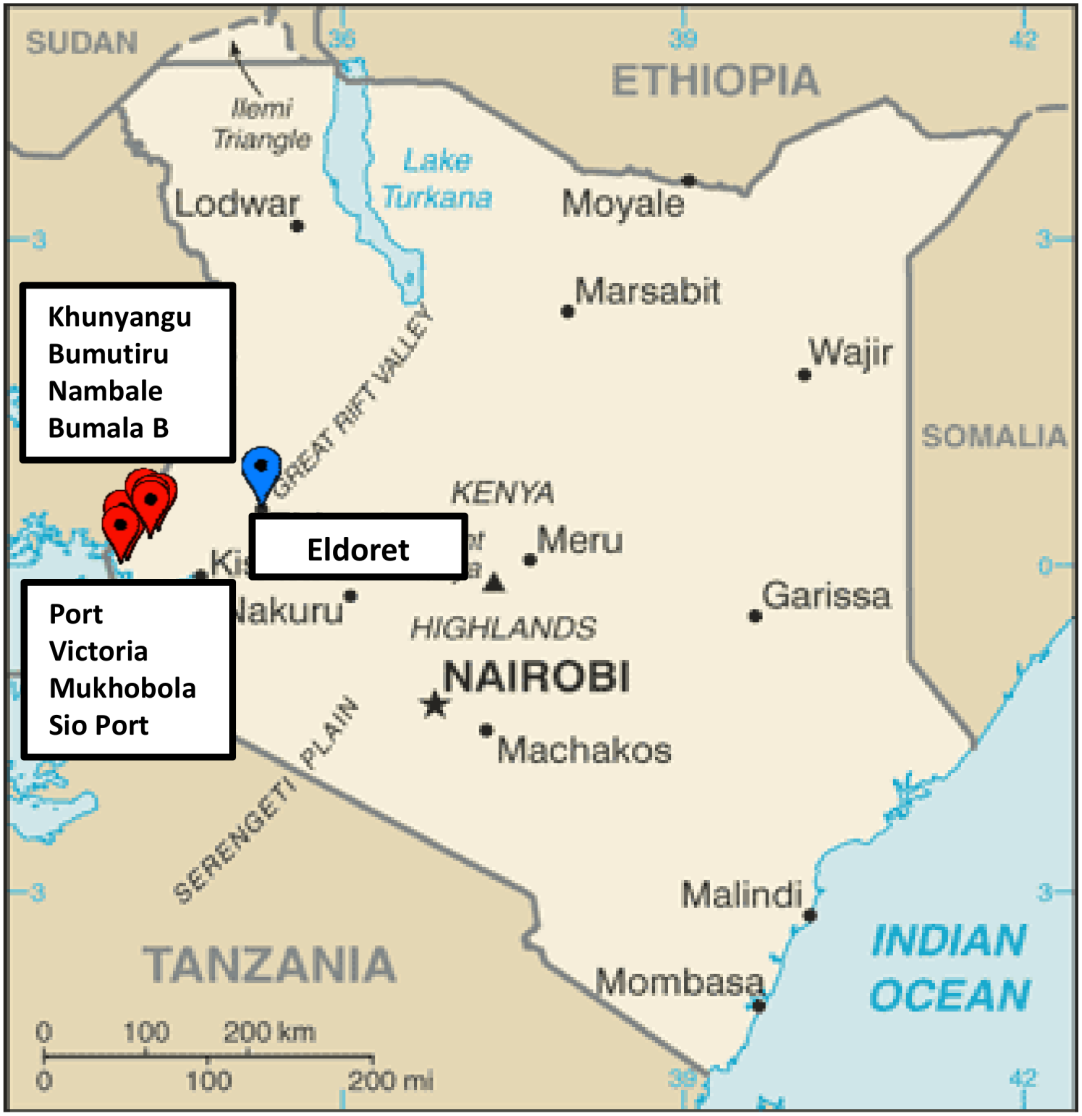
Clinic locations within the urban and rural study settings in western Kenya.

For the rural setting, we worked with health centers in Busia County, a region with 83% of the population living in rural, low population density areas, because of the region’s suspected high burden of drug resistant TB. We initially selected three clinics: Khunyangu and Port Victoria due to their proximity to the Ugandan border, and Mukhobola because the two XDR-TB patients had lived in the town. Each town has a low population density and rural poor patient populations. Additional selection criteria included high patient volume, laboratory capacity, and active case finding in the community. Specifically, each clinic was also a site of the Intensified Case Finding Cough Monitor program (ICF), a community outreach program which trained individuals to lead community awareness efforts, identify symptomatic individuals, and collect and deliver sputum to the laboratory. Because during the first nine months of the 18 month study the patient volume was lower than anticipated, we extended the study site to include four additional clinics: Nambale, Bumala B, Sio Port, and Bumutiru (Fig 1). These clinics were selected based on their geographical proximity, similar patient populations, and similar community outreach efforts to the first three clinics in order to maintain sampling from the same catchment area.

At each clinic, smear microscopy was used to diagnose TB for patients who both sought care at the clinic and who were identified in the community. All treatment-naïve smear positive patients were eligible for screening with culture and DST. Clinical information collected for each patient included demographic, TB contact, health status, and history of illness information.

All treatment-naive patients with confirmed *Mycobacterium tuberculosis* (MTB) were included in the LQAS study. Patients were identified either when they presented to a clinic for evaluation or were screened by cough monitors through active case finding efforts in the community under the ICF program. Children under the age of 15, patients with sputum smear negative TB or extrapulmonary TB, and patients with sputum smear positive TB who had already started treatment were not eligible for the study. Patients screened with culture and DST who had contaminated specimens, nontuberculous mycobacteria, or who were suspected to have *Mycobacterium bovis*, using monoresistance to pyrazinamide as a proxy for *M*. *bovis* diagnosis, were excluded.

### Laboratory testing

Culture and DST for either individual or pooled spot and morning specimens were performed at the Mycobacteria Reference Laboratory (MRL), situated on the grounds of MTRH/AMPATH. All specimens underwent liquid culture with MGIT and identification with capilia. Isolates were tested with MGIT for susceptibility to isoniazid, rifampicin, pyrazinamide, ethambutol, and streptomycin. Specimens from MDR-TB suspects were sent to the CRL of the National TB program for confirmation, as per national guidelines. Quality assurance was conducted on 20% of specimens by the Supranational TB Reference Laboratory at Makerere University in Kampala, Uganda, and all results were confirmed to be accurate.

At MTRH, smear microscopy was performed at the MRL. All specimens were preserved in the refrigerator until patients’ clinical forms were completed one to two days later, at which point the specimens were cultured. At the rural clinics, smear microscopy was conducted on site. This study did not provide external quality assurance (EQA) for smear microscopy, but EQA was conducted quarterly for each clinic by the Kenyan national TB program with all study sites passing inspection. Because the seven rural clinics were up to 125 km from the MRL, specimens were transported to the MRL each week via the AMPATH transport system. To decrease bacterial contamination, specimens were stored in the refrigerator. On the day of transport, specimens were transferred to a cooler, delivered to the MRL, and cultured the day after they were received. Results were available six to seven weeks after specimens were received and were returned to clinicians to guide patients’ treatment plans.

### Sample size for LQAS Classification

In this study, we utilized the LQAS approach to classify areas as having high or low prevalence of MDR-TB. We selected a static, two-way LQAS sampling plan, which uses a predetermined sample size (n) and a corresponding decision rule (d). The decision rule specifies the number of cases in the sample at or above which a region is classified as a high resistance area and below which a region is classified as a low resistance area. The two-way classification design thus specifies that regions are classified into two categories: either having high or low levels of drug resistance. We set the thresholds for classifying a high resistance region at greater than 5% resistance and a low resistance region at less than 1% resistance. These thresholds were selected based on the most recent country-wide MDR-TB burden estimates of for Kenya of 3.1% (0.10–7.0) among treatment naïve patients [10]. We specified an alpha error rate of 10% of misclassifying a high resistance area (false negative: a true high resistance level classified as low) and a beta error rate of 10% of misclassifying a low resistance area (false positive: a true low resistance area classified as high). These errors were set to equal levels reflecting an assumption that each type would be similarly detrimental to TB control efforts.

The sampling plan assumes simple random sampling and uses a binomial model to calculate the sample size (n) and corresponding decision rule (d) based on the thresholds for high and low resistance classification and the tolerance for misclassification errors, as specified above. The resulting sample size was n = 105 and decision rule was d = 3. If three or more patients were diagnosed with MDR-TB out of 105 screened, then the region would be classified as high resistance and if fewer than 3, then the region was classified as low resistance.

### Ethics Statement

This project received approval from the Institutional Review Boards at Moi University School of Medicine/AMPATH, Alpert School of Medicine at Brown University, and Partners IRB (Boston). A waiver of individual informed consent was approved because the project provided drug sensitivity testing for patients, the standard of TB care, and data were then de-identified and analyzed anonymously.

## Results

Using a static, two-way LQAS classification plan, we assessed levels of resistance in one urban and one rural setting in western Kenya from October 2011 to May 2013. For the urban setting, sampling was performed at the outpatient TB clinic at Moi Teaching and Referral Hospital in Eldoret, Kenya. For the rural site, sampling was performed at seven clinics in the towns of Khunyangu, Port Victoria, Mukhobola, Nambale, Bumala B, Sio Port, and Bumutiru. Approximately 40% of patients screened within the urban site and 44% of patients within the rural site were HIV positive.

Out of 105 patients screened with culture and DST in each setting, two patients were diagnosed with MDR-TB in the urban setting and one patient was diagnosed with MDR-TB in the rural setting. Because the number of cases in each setting was below the predetermined decision rule of 3 cases, the study classified both regions as having low levels of TB drug resistance.

Of the 210 treatment-naive sputum smear positive patients screened, 27 other patients were diagnosed with a variety of mono- and poly- resistant strains (Table 1).

**Table 1:** Drug resistance patterns among patients with sputum smear positive TB within the urban and rural study settings in western Kenya.

	Patients		
Drug Resistance Pattern	Urban site	Rural site	All
	n = 105	n = 105	n = 210
Pan-susceptible	89	91	180
*Mono resistant to*			
Isoniazid	5	5	10
Rifampicin	1	0	1
Total	6	5	11
*Poly resistant to*			
Isoniazid	4	6	10
Rifampicin	0	0	0
Total	4	6	10
Other resistance (without Isoniazid or Rifampicin)	4	2	6
MDR-TB	2	1	3
Classification of Resistance	Low	Low	N/A

## Discussion

Population-wide benefits of drug resistance surveillance include the ability to estimate resource requirements for second-line treatment, evaluate trends in resistance, and assess the effects of TB control efforts. Most existing drug resistance surveys rely on DST among random samples of diagnosed TB cases countrywide to produce estimates of the fraction of cases with MDR-TB [3,11,12]. Most are conducted by screening treatment-naïve smear positive patients who seek diagnosis at a clinic. Depending on laboratory capacity, sputum samples may be tested on site or may be shipped to reference laboratory. While these surveys provide valuable nationwide assessments, they are subject to a variety of limitations. In addition to being logistically and financially challenging, they are not designed to provide information on the variation of resistance that may exist between different geographical regions. Such localized information may be important for national TB programs to design more targeted interventions for identified high burden geographical regions and to optimally allocate limited resources. Further, because these surveys are often conducted infrequently, using a method to identify hotspots as suspicion arises may supplement country-wide surveys and allow for more rapid responses [12].

In this study, we employed LQAS as an alternative approach for resistance surveillance to identify potential geographic heterogeneity in the risk of resistance among treatment-naïve cases. Instead of aiming to produce precise estimates of the fraction of cases with resistance, the goal of LQAS is to classify the level of resistance as high or low as defined by pre-specified thresholds. LQAS approaches have been previously used for surveillance of HIV, trachoma, and schistosomiasis [13–17]. In its simplest two-way classification form, as used here, the LQAS methodology is designed to classify regions as areas of either low or high resistance, thereby identifying specific regions requiring intervention [7,18].

While we previously demonstrated the potential utility of LQAS for uncovering geographic heterogeneity from reported survey data [19], this study represents the first attempt to prospectively use LQAS to assess whether local risks of resistance exceeded a pre-determined threshold. Rather than applying LQAS to published country-wide data to identify regions of resistance, we conducted a targeted drug resistance survey using LQAS within two settings of interest. Our decision to use LQAS to classify levels of local resistance was motivated by recent concern about suspected high levels of resistance in rural districts of western Kenya. Our results suggest that drug resistance currently remains low in each of the settings evaluated.

Conducting a prospective LQAS study targeting specific regions represents a two-stage screening model. The first stage is identifying regions with suspected high levels of resistance. Criteria for suspecting elevated resistance may include, among others, high levels of treatment interruption, high levels of re-treatment cases, reports of treatment failure, and reports or case studies documenting resistance (as was the case for this study). The second stage is applying LQAS to classify the levels of resistance in these regions. We note that the suspected higher prevalence in targeted regions may also function to increase the positive predictive value and decrease the negative predictive value of the testing methodology.

As compared to countrywide surveys, targeted LQAS typically costs less than the average national survey price of $250,000-$300,000, given that fewer resources are needed to evaluate a smaller sample size [4], and typically has more rapid completion times. As a result, LQAS may offer TB control programs an alternative approach to evaluate levels of resistance in suspected localized hotspots or to investigate emerging resistance in the time between conducting larger surveys. Information about high or low levels of resistance may help inform programs as to the need to re-evaluate policies. Such policies include free provision of DST only for failing or relapsed patients rather than for all TB patients and all contacts of patients diagnosed with drug resistant TB as well as the use of alternative initial empiric treatment regimens for TB patients pending further information in areas where primary drug resistance is found to be at a high level. Although this study did not speed the survey completion time, the study provided information about a specific area with suspected high levels of resistance during a time period when the next country-wide survey was not yet being conducted and may not have included the potential hotspot as a site of sampling. Findings were shared with the national TB program, which was prepared to target greater resources to both sites if they had been classified as having high resistance.

While we believe this study helps demonstrate how LQAS may serve as a tool for timely assessment of local variation of resistance, we note that several limitations may affect the results of this study. First, the survey was restricted to sputum smear positive patients during a time period of 18 months. Because patients co-infected with TB/HIV are more likely to present with sputum smear negative TB, our results may not be generalizable to this important patient subgroup. While the association between HIV and MDR-TB is currently unclear, it is possible that the study underestimated resistance by preferentially excluding TB/HIV co-infected patients [12,20]. Another limitation was the need to expand the catchment area for the rural setting due to lower than expected case detection at the original sites. While each additional site was within 30 km of an existing site and had a similar patient population, the extended area may not have been as representative of the suspected region of high resistance.

Further, we encountered several logistical challenges that may have compromised sampling and thus may have biased results. For example, flooding at the Mukhobola clinic (rural setting), resulted in a month long closure to enrollment. In the rural clinics, several occurrences of supply shortages, inadequate staffing, and prolonged turn around time for routine microscopy limited laboratories’ capacity for case detection. Maintaining a cold chain during specimen transport was also an obstacle in that a number of specimens were contaminated, up to 11% per clinic, by the time they arrived at the MRL. This problem is common to surveys regardless of design that do not conduct DST on site, suggesting that rapid, on site resistance testing may improve sampling. The validity of our results assume that each of these issues resulted in potentially eligible patients being omitted from the study at random with respect to drug resistance, an assumption which is reasonable, but not testable with the available data.

For future studies, some of the limitations that we faced may be mitigated with recent investments into more rapid diagnostics. Kenya, along with many other countries in the African Region, have recently committed to scaling up the use of molecular technologies such as Xpert MTB/RIF to rapidly diagnose TB and drug resistance [21,22]. Such advances in laboratory capacity could allow for the inclusion of smear negative patients, providing for potentially more representative sampling. In addition, expanded laboratory capacity with rapid diagnostics could help address logistical challenges, such as speed of diagnosis and reduced contamination if samples are tested on site or at more proximal facilities, thereby expediting sampling and potential interventions in the future.

Another consideration for future LQAS designs includes appropriate designation of thresholds and acceptable levels for misclassification errors. We selected a static two-way classification approach with 1% low and 5% high thresholds based upon resistance estimates from Kenya’s last national survey and alpha and beta error rates of 10%. Study design, including the determination of two- or three-way classification (in which a three-way classification would classify regions as high, medium, and low) and choice of thresholds and tolerance for error, should be guided by previous surveys, which might indicate expected levels of resistance, and by policy considerations of which interventions will be invoked if specified thresholds are exceeded [7,19]. Alternatively, surveys could be designed to classify other resistance phenotypes, but this would most likely necessitate specifying different appropriate classification thresholds specific to those phenotypes; for example, if the goal was to classify based on isoniazid resistance, the thresholds selected would likely be substantially higher than those we used for classifying MDR-TB. Overall, future resistance surveys in this region can build off of the data gathered during this survey to guide updated choices of study design and threshold levels.

## Conclusion

This LQAS study suggests that both regions investigated have low levels of resistance. Further drug resistance surveillance using LQAS, particularly after addressing logistical barriers, may help identify the levels and geographical distribution of drug resistance in Kenya and may have applications in other countries in the African Region facing similar resource constraints.

## Supporting Information

S1 Dataset Drug resistant patterns among patients with sputum smear positive TB within the urban study setting (XLSX)

S2 Dataset Drug resistant patterns among patients with sputum smear positive TB within the rural study setting (XLSX)
